# Correlation Between TCF7^+^ T Cells and Prognosis of Patients With Oral Squamous Cell Carcinoma

**DOI:** 10.3389/fonc.2022.782058

**Published:** 2022-03-08

**Authors:** Haixu Rong, Tingting Cai, Yu Peng, Xiaojuan Wang, Tianjun Lan, Zhanpeng Ou, Ling Qiu, Qunxing Li, Lizao Zhang, Fan Wu, Hsinyu Lin, Siqi Ren, Zitian Li, Song Fan, Jinsong Li

**Affiliations:** ^1^ Guangdong Provincial Key Laboratory of Malignant Tumor Epigenetics and Gene Regulation of Sun Yat-sen Memorial Hospital, Guangzhou, China; ^2^ Department of Oral and Maxillofacial Surgery, Sun Yat-sen Memorial Hospital of Sun Yat-sen University, Guangzhou, China; ^3^ The Stomatology Department of The First Affiliated Hospital, Medical College of Shantou University, Shantou, China; ^4^ School of Stomatology, Jilin University, Changchun, China

**Keywords:** oral squamous cell carcinoma, TCF7/TCF1, survival analysis, Cox regression, prognosis, nomogram

## Abstract

**Objective:**

To investigate whether TCF7^+^ T cells constitute an important factor to improve the existing postoperative prediction model for patients with oral squamous cell carcinoma.

**Method:**

TCF7^+^ T cells were detected in the tissues of 167 OSCC patients by multiplex immunofluorescence. The percentage of TCF7^+^ T cells was transformed into a dichotomous variable, combined with the clinicopathological data for the OSCC patients, and then subjected to univariate and multivariate analyses. The derived independent predictors were then incorporated into risk models to analyze their relationship with the prognosis of patients.

**Results:**

The high TCF7^+^ group had a better prognosis than the low TCF7^+^ group (OS: *p*<0.001; RFS: *p*<0.001). Univariate and multivariate analyses showed that TCF7^+^ T cells serve as an independent predictor of OSCC (univariate/multivariate analysis: *p*<0.001). In Cox risk progression models, inclusion of the TCF7^+^ T cell percentage improved the predictive accuracy of Grade and TNM stage (Grade-OS/RFS: *p*<0.001; TNM-OS/RFS: *p*<0.001; TNM+Grade-OS: *p*<0.001, TNM+Grade-RFS: *p*=0.004). Inclusion of the TCF7^+^ T cell percentage improved the clinical utility.

**Conclusions:**

TCF7^+^ T cells can act as an independent predictor for postoperative OSCC patients. The inclusion of TCF7^+^ T cells improved the predictive accuracy and clinical utility of the nomograms to different degrees.

## Introduction

Oral squamous cell carcinoma (OSCC) is a common tumor worldwide. In 2020, an estimated 377,713 cases of oral cancer occurred, with an estimated 177,757 deaths and male dominance, making it the 8th most common tumor in men (1). Despite progress in the treatment of solid tumors with emerging immunotherapies (2), significant therapeutic effects are lacking in nonresponders (3, 4), and immune-related adverse events may occur (5, 6). Therefore, to expand the therapeutic population of immunotherapy, improve the response rates and increase the duration of enduring remission, the search for new markers has become urgent.

Studies have shown a stronger antitumor response when more self-renewing, expanding and persistent T cells are present within the tumor (7, 8). T cell factor 1 (TCF1) is a transcription factor of the typical Wnt signaling pathway encoded by the TCF7 gene (9). The critical role of TCF1 in T cell differentiation and memory formation is widely recognized. For example, Moshe et al. (10) reported high expression of markers such as PLAC8, LTB, LY9, SELL, TCF7, and CCR7 in T cells in response to anti-PD-1 treatment. Additionally, TCF7^+^ (TCF1^+^) T cells (hereafter referred to as TCF7^+^ T cells) amplify immune responses and improve the response to immunotherapy in cancer. The reason may be that T cells appear to have a stem cell-like ability to expand, differentiate and self-renew by expressing large amounts of TCF7 (11). Therefore, Moshe et al. suggested that TCF7^+^ T cells may improve the prognosis of immune checkpoint inhibitor therapy for melanoma. However, the prognosis of TCF7^+^ T cells in OSCC is unclear.

In this study, multiplex immunofluorescence was used to detect the expression of TCF7^+^ T cells in OSCC, combined with the clinicopathological data for 167 OSCC patients. After univariate analysis and multivariate analysis, the variables with statistical significance were transformed into a Cox risk prediction model to elucidate the potential of TCF7^+^ T cells as a new marker valuable reference for predicting OSCC patient prognosis.

## Materials and Methods

### TCGA and Survival Data

The transcriptomic data and clinical information shown in the **Supplemental Figures** were obtained from the TCGA repository at GDC (https://portal.gdc.cancer.gov/). mRNA expression screening of OSCC patients was performed according to NCCN Guidelines Version 2.2021. The expression of CD3 and TCF7 transcripts, survival time and survival status were included in the downloaded survival data. To obtain reliable and ideal grouping, each gene was divided into high- and low-expression groups using the R packages “survminer” and “maxstat”; *p value*s were calculated using the log-rank test.

### Data Collection and Patient Information

The sample size followed the principle of 20 EPV (Event Per Variable) (12), and 167 paraffin tissue specimens from surgical resection at the Department of Oral and Maxillofacial Surgery, Sun Yat-sen Memorial Hospital, Sun Yat-sen University were collected from January 2015 to December 2019. All the patients met the following criteria: (a) a first pathologic diagnosis of OSCC; (b) no radiotherapy or chemotherapy before surgery; (c) preserved postoperative wax blocks; (d) available complete electronic medical records, histological pathology reports and follow-up records. Clinicopathological factors, including age, sex, alcohol consumption, tobacco factor, primary site location, T stage, lymph node metastasis, TNM stage, histological grade, CD3^+^ T cell expression, and CD3^+^TCF7^+^ T cell expression, were collected. The clinical stages and differentiation degrees of the tumor were classified based on TNM classification of the AJCC 7th edition and WHO grade classification, respectively. The 167 patients were followed up postoperatively every 3 months for 1 year and every 6 months from the second year for 5 years. Written informed consent was obtained from each patient participating in the study.

### Multiplexed Immunofluorescence and Evaluation

All the paraffin tissue specimens were fixed with 4% formaldehyde, embedded in conventional paraffin, serially sectioned at approximately 4 μm thickness, and dewaxed. EDTA alkaline antigen retrieval was performed, and the samples were blocked with goat serum. After incubation with the primary antibody, the slides were washed with TBST for 3 min, incubated with the secondary antibody (goat anti-mouse HRB, 10 min), washed with TBST for 3 min and incubated with a fluorescent dye (Panovue). The above process was repeated for additional markers, after which DAPI was added dropwise; after staining, an anti-quenching agent was added dropwise, and coverslips were sealed. The incubation conditions were as follows: TCF7 (cst, rabbit monoclonal antibody, 1:200, first cycle), CD3 (Abcam, rabbit monoclonal antibody, 1:100, second cycle), and DAPI (1:200). The tissue sections were scanned using the Akoya Vectra Polaris system (1.0.10; PerkinElmer, Inc), and images were analyzed using inForm software (2.4.6; PerkinElmer, Inc). The results were evaluated as follows: four fields of view were selected arbitrarily for each patient’s section, the percentage of CD3^+^ TCF7^+^ T cells among total CD3^+^ T cells in each field of view was obtained, and the sum was averaged as the percentage of TCF7^+^ T cells in the tumor. The optimal cutoff percentage values of TCF7^+^ T cells were divided in X-tile 3.6.1 (Yale University, New Haven, Connecticut, USA) software (13) based on OS and RFS.

### Statistical Analysis

Statistical analysis was performed using SPSS 25.0 (SPSS, Inc., Chicago, IL, USA) and R (R Foundation for Statistical Computing, Beijing, China). In the baseline table, the measures were expressed as means ± standard deviation, and *χ²* test was used for comparisons between groups. Cox regression was employed for both univariate and multivariate analyses. The *p value* was determined by the *Benjamini & Hochberg* (BH) method for multiple testing correction. *p*<0.05 was considered a statistically significant difference. The statistically significant variables in univariate analysis were subjected to multivariate analysis, and the variables ultimately selected were used as variables in the nomogram. Nomograms and calibration curves were plotted using R software (rms package, survivor package). Regarding the performance of the predictive model, the C-index was applied to evaluate the discrimination of different models, where the C-index was compared using the *compareC* function. Calibration curves were used to assess calibration of the model under 1000 resamplings (B=1000). We used decision curve analysis to compare clinical utility among the models at 1, 3 and 5 years.

## Results

### Expression of TCF7^+^ T Cells in OSCC Tissues and Its Correlation With Clinicopathological Features

We first estimated the prognostic value of CD3^+^TCF7^+^ T cells in OSCC patients at the transcriptional level. The transcriptome data for 212 OSCC patients and their survival information were downloaded from TCGA. The patients were divided into a CD3^hi^ TCF7^hi^ (DH) group, CD3^low^ TCF7^low^ (DL) group, CD3^hi^ TCF7^low^ (CD3^hi^) group, and CD3^low^ TCF7^hi^ (TCF7^hi^) group based on transcript levels. No significant difference (*p*=0.160) was found in the OS rates among the four groups (**Supplementary Figure 1A**). However, statistical significance (*p*=0.041) was shown for the DH group and DL group (**Supplementary Figure 1B**), indicating that the DH group had a better prognosis based on transcriptional data.

**Figure 1:**
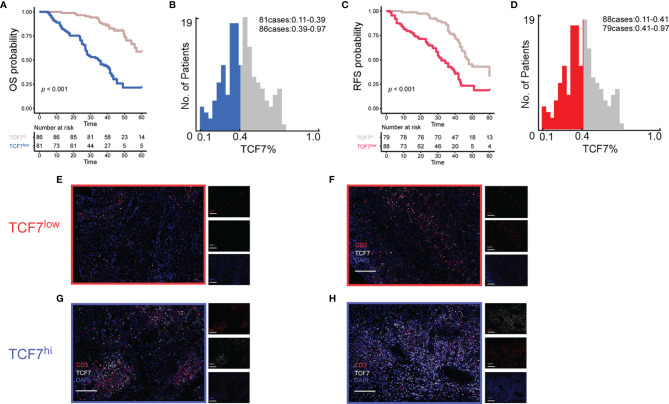
A total of 167 patients were classified into the TCF7^+^ T cell low expression group and TCF7^+^ T cell high expression group based on optimal cutoff values of 0.39 **(B)** and 0.41 **(D)** for OS **(A)** and RFS **(C)**, respectively, using X-tile software. Representative multiplex immunofluorescence results plots were selected in each of the two groups to demonstrate the expression of TCF7^+^ T cells in the low expression group **(E, F)** and high expression group **(G, H)**. The markers of fluorescence used were CD3 (red), TCF7 (white), and DAPI (blue). Magnification, 20×; white scale bar, 100 μm; OS, overall survival; RFS, recurrence-free survival.

To test whether TCF7^+^ T cells also influence the prognosis of OSCC patients, the number of TCF7^+^ T cells was assessed by multiplex immunofluorescence analysis in 167 OSCC patients (**Figure 1**). The proportion of TCF7^+^ T cells to CD3+ T cells was used as a continuous variable. The 167 patients were divided into a TCF7^+^ T cell low expression group (hereafter referred to as the TCF7^low^ group) (**Figures 1E, F**) and a TCF7^+^ T cell^hi^ expression group (hereafter referred to as the TCF7^hi^ group) according to the results calculated by X-tile software (**Figures 1A**–**D, G, H**). The cutoff value in the OS cohort was 0.39, with 81 patients assigned to the TCF7^low^ group (**Figure 1B**); the cutoff value in the RFS cohort was 0.41, with 88 patients assigned to the TCF7^low^ group (**Figure 1D**). Based on both OS and RFS survival curves, the TCF7^hi^ group had a more favorable prognosis than the TCF7^low^ group (**Figures 1A, C**). Associations between the TCF7^hi^ and TCF7^low^ groups in the OS cohort and RFS cohort and clinicopathological characteristics are summarized in **Table 1**. TCF7^+^ T cells were associated with T stage, lymph node metastasis and TNM stage, with statistically significant differences (*p*<0.05), but not with sex, age, tobacco, alcohol, histological grade, or CD3 density (*p*> 0.05). Clinicopathological baseline tables for the TCF7^hi^ and TCF7^low^ groups were established (**Table 1**).

**Table 1:** Clinicopathological characteristics of OSCC patients according to TCF7 expression.

Characteristic	Overall (n=167)	OS			RFS		
		TCF7^hi^ (n=86)	TCF7^low^ (n=81)	*p value* ^b^	TCF7^hi^ (n=79)	TCF7^low^ (n=88)	*p value* ^b^
**Age, y (mean ± SD)**	53.71 ± 12.71	53.86 ± 12.08	53.54 ± 13.41	0.161	54.16 ± 11.80	53.30 ± 13.53	0.660
**Sex**							
**Male**	98 (58.68%)	51 (59.30%)	47 (58.02%)		46 (58.23%)	52 (59.09%)	
**Female**	69 (41.32%)	35 (40.70%)	34 (41.98%)	0.992	33 (41.77%)	36 (40.91%)	0.999
**Alcohol**							
**No**	97 (58.08%)	52 (60.47%)	45 (55.56%)		47 (59.49%)	50 (56.82%)	
**Yes**	70 (41.92%)	34 (39.53%)	36 (44.44%)	0.627	32 (40.51%)	38 (43.18%)	0.847
**Tobacco**							
**No**	104 (62.28%)	52 (60.47%)	52 (64.20%)		48 (60.76%)	56 (63.64%)	
**Yes**	63 (37.72%)	34 (39.53%)	29 (35.80%)	0.736	31 (39.24%)	32 (36.36%)	0.823
**Primary tumor site**							
**Oral tongue**	133 (79.64%)	70 (81.40%)	63 (77.78%)		65 (82.28%)	68 (77.27%)	
**Gingiva**	12 (7.19%)	8 (9.30%)	4 (4.94%)		8 (10.13%)	4 (4.55%)	
**Buccal mucosa**	7 (4.19%)	1 (1.16%)	6 (7.41%)		1 (1.27%)	6 (6.82%)	
**Hard palate**	5 (2.99%)	2 (2.33%)	3 (3.70%)		1 (1.27%)	4 (4.55%)	
**Floor of mouth**	10 (5.99%)	5 (5.81%)	5 (6.17%)	0.262	4 (5.06%)	6 (6.82%)	0.167
**T-stage^a^**							
**T1**	50 (29.94%)	35 (40.70%)	15 (18.52%)		34 (43.04%)	16 (18.18%)	
**T2**	42 (25.15%)	22 (25.58%)	20 (24.69%)		21 (26.58%)	21 (23.86%)	
**T3**	75 (44.91%)	29 (33.72%)	46 (56.79%)		24 (30.38%)	51 (57.95%)	
**T4**	0	0	0	0.003	0	0	<0.001
**N-stage** **^a^**							
**N0**	73 (43.71%)	51 (59.30%)	22 (27.16%)		49 (62.03%)	24 (27.27%)	
**N1**	30 (17.96%)	14 (16.28%)	16 (19.75%)		13 (16.64%)	17 (19.32%)	
**N2a**	6 (3.59%)	3 (3.49%)	3 (3.7%)		3 (3.80%)	3 (3.41%)	
**N2b**	57 (34.13%)	18 (20.93%)	39 (48.15%)		14 (17.72%)	43 (48.86%)	
**N2c**	1 (0.60%)	0	1 (1.23%)	<0.001	0	1 (1.14%)	<0.001
**TNM stage** **^a^**							
**I**	40 (23.95%)	31 (36.05%)	9 (11.11%)		29 (36.71%)	11 (12.50%)	
**II**	29 (17.37%)	18 (20.93%)	11 (13.28%)		18 (22.78%)	11 (12.50%)	
**III**	31 (18.56%)	15 (17.44%)	16 (19.75%)		14 (17.72%)	17 (19.32%)	
**IV**	67 (40.12%)	22 (25.58%)	45 (55.56%)	<0.001	18 (22.78%)	49 (55.68%)	<0.001
**Histologic grade**							
**Well**	72 (43.11%)	40 (46.51%)	32 (39.51%)		36 (45.57%)	36 (40.91%)	
**Poor**	95 (56.89%)	46 (53.49%)	49 (60.49%)	0.449	43 (54.43%)	52 (59.09%)	0.652
**CD3**	1311 ± 946	1219 ± 837	1408 ± 1047	0.197	1177 ± 818	1431 ± 1038	0.084

a American Joint Committee on Cancer,7^th^ Edition staging.

b Fisher’s exact test or chi-squared test was used to examine the correlation between TCF7 expression and clinicopathological characteristics in 167 patients with OSCC.

### TCF7^hi^ Predicts a Good Prognosis for OSCC Patients Regarding OS and RFS

We performed univariate and multivariate analyses to further assess whether the TCF7^+^ T cell proportion is an independent predictor of OS and RFS. Univariate analysis showed (**Table 2**) that the TCF7^+^ T cell percentage as a continuous variable was a risk factor for OS and RFS (OS: HR=0.013, 95% CI=0.002 to 0.074, *p*<0.001; RFS: HR=0.041, 95% CI=0.009 to 0.185, *p*<0.001) and that T stage (T2-OS: HR=2.187, 95% CI=0.814 to 5.872, *p*=0.185; T2-RFS: HR=1.056, 95% CI=0.572 to 1.950, *p*=0.919; T3-OS: HR=8.621, 95% CI=3.709 to 20.039, *p*<0.001; T3-RFS: HR=2.645, 95% CI=1.592 to 4.397), lymph node metastasis (N1-OS: HR=4.510, 95% CI=2.077 to 9.792, *p*<0.001; N1-RFS: HR=3.548, 95% CI=1.972 to 6.382, *p*<0.001; N2a-OS: HR=7.789, 95% CI=2.718 to 22.327, *p*<0.001; N2a-RFS: HR=5.077, 95% CI=2.064 to 12.490, *p*<0.001; N2b-OS: HR=7.770, 95% CI=4.065 to 14.854, *p*< 0.001; N2b-RFS: HR=4.770, 95% CI=2.881 to 7.900, *p*<0.001; N2c-OS: HR=9.796, 95% CI=1.261 to 76.104, *p*=0.0517, N2c-RFS: HR<0.001, 95% CI=0 to infinity, *p*=0.996), TNM stage (II-OS: HR=1.695, 95% CI=0.401 to 7.155, *p*=0.541; II-RFS: HR=0.822, 95% CI=0.364 to 1.857, *p*=0.113), histological grade (OS: HR=0.471, 95% CI=0.286 to 0.776, *p*=0.006; RFS: HR=0.564, 95% CI=0.367 to 0.868, *p*=0.018) were also risk factors for OS and RFS. Additionally, the percentage of TCF7^+^ T cells was transformed into a dichotomous variable and then included in multivariate analysis (**Table 3**). Thus, the TCF7^+^ T cell expression level was a risk factor for OS and RFS (OS: HR=0.235, 95% CI=0.133 to 0.414, *p*<0.001; RFS: HR=2.661, 95% CI=1.675 to 4.227, *p*<0.001). In multivariate analysis, TNM stage (OS: HR=8.539, 95% CI=2.144 to 34.013, *p*=0.004; RFS: HR=4.992, 95% CI=1.054 to 23.649, *p*=0.086) and histological grade (OS: HR=0.408, 95% CI=0.246 to 0.679, *p*=0.002; RFS: HR=0.469, 95% CI=0.303 to 0.724, *p*=0.001) were risk factors for OS and RFS.

**Table 2:** Univariate analysis of factors associated with overall survival.

Univariate analysis	OS (n=167)		RFS (n=158)	
Variable	HR (95% CI)	p value*	HR (95% CI)	p value*
**Sex**				
**Male vs. Female**	1.032 (0.649-1.643)	0.893	1.058 (0.702-1.594)	0.900
**Age**				
**<50 vs. >50**	1.285 (0.809-2.041)	0.354	1.232 (0.818-1.855)	0.424
**Tobacco**				
**Yes vs. No**	0.726 (0.449-1.178)	0.260	0.799 (0.526-1.214)	0.424
**Alcohol**				
**Yes vs. No**	1.127 (0.712-1.78)	0.651	1.279 (0.854-1.915)	0.371
**T stage**				
**T1**	Ref.		Ref.	
**T2**	2.187 (0.814-5.872)	0.185	1.056 (0.572-1.950)	0.919
**T3**	8.621 (3.709-20.039)	<0.001	2.645 (1.592-4.397)	<0.001
**Node metastasis**				
**N0**	Ref.		Ref.	
**N1**	4.510 (2/077-9.792)	<0.001	3.548 (1.972-6.382)	<0.001
**N2a**	7.789 (2.718-22.327)	<0.001	5.077 (2.064-12.490)	<0.001
**N2b**	7.770 (4.065-14.854)	<0.001	4.770 (2.881-7.900)	<0.001
**N2c**	9.796 (1.261-76.104)	0.0517	<0.001 (-)	0.996
**TNM stage**				
**I**	Ref.		Ref.	
**II**	1.695 (0.401-7.155)	0.541	0.822 (0.364-1.857)	0.113
**III**	9.079 (1.626-31.380)	0.001	3.289 (1.632-6.624)	0.002
**IV**	15.450 (4.815-49.573)	<0.001	4.645 (2.510-8.609)	<0.001
**Histologic Grade**				
**Well vs. Poor**	0.471 (0.286-0.776)	0.006	0.564 (0.367-0.868)	0.018
**CD3**	1.000 (1.000-1.000)	0.185	1.000 (0.999-1.000)	0.897
**TCF7**	0.013 (0.002-0.074)	<0.001	0.041 (0.009-0.185)	<0.001

HR, hazard ratio; CI, confidence interval.

p value*: Multiple test correction by the “Benjamini & Hochberg” (BH) method.

**Table 3:** Multivariate analysis of factors associated with overall survival.

Multivariate analysis	OS (n=167)		RFS (n=167)	
Variable	HR (95% CI)	p value*	HR (95% CI)	p value*
**T stage**				
**T1**	Ref.		Ref.	
**T2**	2.037 (0.740-5.604)	0.202	1.009 (0.534-1.908)	0.977
**T3**	2.597 (1.070-6.303)	0.053	0.773 (0.432-1.384)	0.581
**Node metastasis**				
**N0 vs. N+**	1.284 (0.458-3.596)	0.635	0.924 (0.223-3.837)	0.977
**TNM stage**				
**I +II vs. III+IV**	8.539 (2.144-34.013)	0.004	4.992 (1.054-23.649)	0.086
**Histologic Grade**				
**Well vs. Poor-**	0.408 (0.246-0.679)	0.002	0.469 (0.303-0.724)	0.001
**TCF7**				
**Low vs. High**	0.235 (0.133-0.414)	<0.001	2.661 (1.675-4.227)	<0.001

HR, hazard ratio; CI, confidence interval.

p value*: Multiple test correction by the “Benjamini & Hochberg” (BH) method.

### Survival Analysis of TCF7^+^ T Cell Expression in OSCC Patients With Different Risk Levels

**Figure 2:**
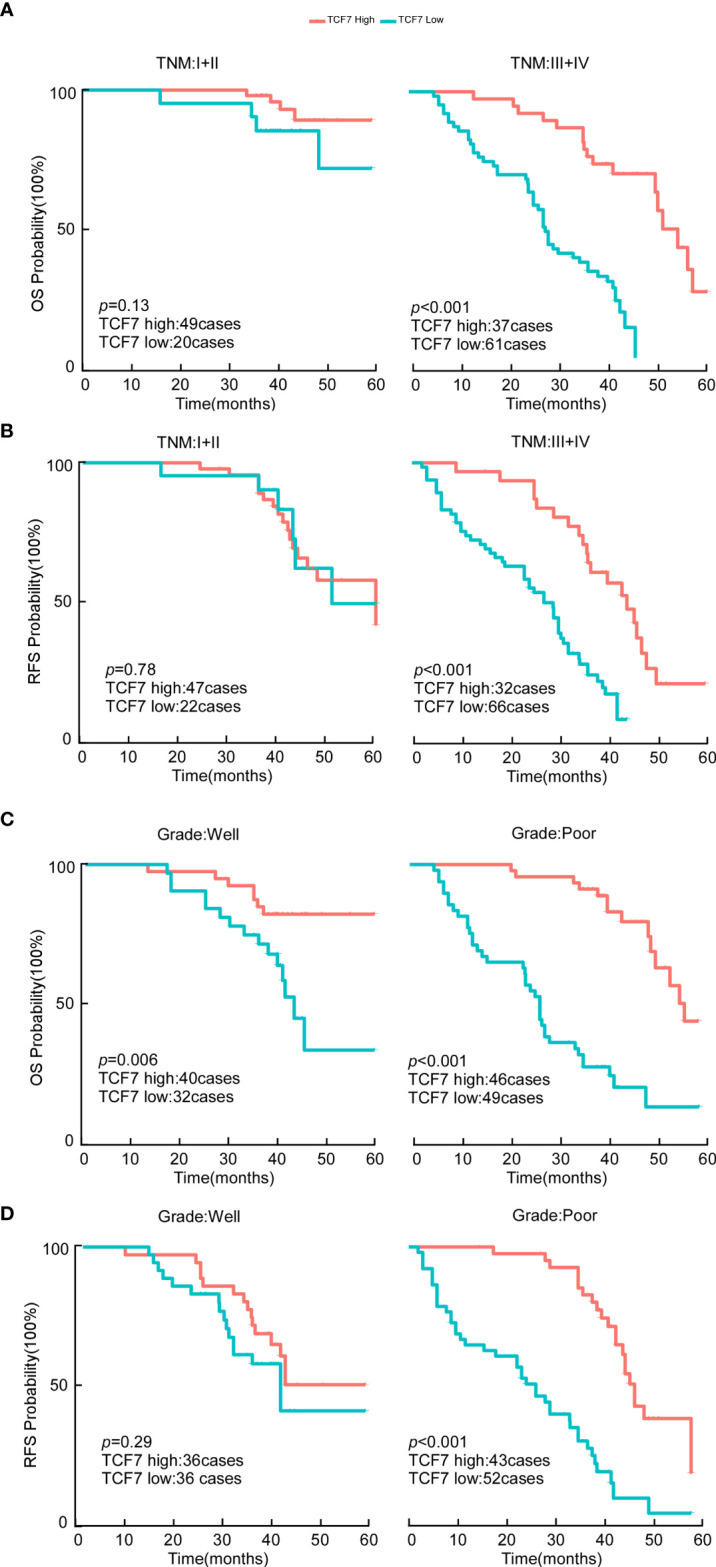
OS and RFS analysis of OSCC patients in the TCF7^+^ T cell high expression group versus the TCF7^+^ T cell low expression group. **(A)** Kaplan–Meier analysis of OS in the TNM low-risk group (n=69) and high-risk group (n=98). **(B)** Kaplan–Meier analysis of RFS in the TNM low-risk group (n=69) and high-risk group (n=98). **(C)** Kaplan–Meier analysis of OS in the histological grade low-risk group (n=72) and high-risk group (n=95). **(D)** Kaplan–Meier analysis of RFS in the histological grade low-risk group (n=72) and high-risk group (n=95). The *p* values of survival curves were calculated by the log-rank test. *p*<0.05 was considered to indicate significance. OS, overall survival; RFS, recurrence-free survival.

Patients were divided into two clinical risk levels by TNM stage: TNM (I+II) and TNM (III+IV) (**Figure 2**). Differences in the percentage of TCF7^+^ T cells regarding OS and RFS were analyzed separately. Although differences in TNM (I+II) between the TCF7^hi^ group and TCF7^low^ group were not statistically significant for OS or RFS (OS: *p*=0.130, **Figure 2A**; RFS: *p*=0.780, **Figure 2B**), differences between the TCF7^hi^ group and TCF7^low^ group for TNM (III+IV) were statistically significant (OS: *p*<0.001, **Figure 2A**; RFS: *p*<0.001, **Figure 2B**). Similarly, histological grade classified patients into two pathological risk levels: a well-differentiated group (well) and a poorly differentiated group (poor). In the well-differentiated group, differences in OS and RFS between the TCF7^hi^ and TCF7^low^ groups were not statistically significant (Well-OS: *p*=0.006, **Figure 2C**; Well-RFS: *p*=0.290, **Figure 2D**). However, in the poorly differentiated group, differences in OS and RFS were both statistically significant (Poor-OS: *p*<0.001, **Figure 2C**; Poor-RFS: *p*<0.001, **Figure 2D**).

### TCF7^+^ T Cell Expression Improves the Predictive Accuracy of Existing Models

In a previous analysis, TCF7^+^ T cell expression could be used as an independent predictor of postoperative OSCC; this variable alone was included in the Cox risk regression model, which calculated a C-index=0.701 (95% CI=0.654 to 0.748) for TCF7^+^ T cell expression for OS and a C-index=0.654 (95% CI=0.610 to 0.698) for RFS, (**Table 4**), suggesting that the model has good predictive accuracy. Next, significantly different variables (TNM stage, histological grade, and TCF7^+^ T-cell expression) were obtained from the results of multivariate analysis. Different Cox risk regression models using the C-index as a measure were established to investigate whether inclusion of the TCF7^+^ T cell percentage enhances the predictive accuracy of the TNM stage and histological grade. Inclusion of the TCF7^+^ T cell percentage improved the prediction accuracy of the TNM stage and histological grade (**Table 4**). The variable grade also improved the C-index from 0.594 (0.539–0.649) to 0.746 (0.690–0.802) for OS (*p*<0.001) and from 0.564 (0.511–0.617) to 0.686 (0.631–0.740) for RFS (*p*<0.001) after including the TCF7^+^ T cell percentage (*p*<0.001). The variable TNM stage increased the C-index from 0.732 (0.685–0.779) to 0.798 (0.752–0.838) for OS (*p*<0.001) and from 0.690 (0.646–0.734) to 0.740 (0.695–0.785) (*p*<0.001) for PFS. In TNM+Grade multivariate analysis, the C-index increased from 0.765 (0.715–0.815) to 0.820 (0.778–0.862) for OS (*p*<0.001) and from 0.728 (0.678–0.777) to 0.758 (0.711–0.805) for RFS after including the TCF7^+^ T cell percentage (*p* = 0.004).

**Table 4:** Comparison of the predictive accuracy of the prognostic models.

Model	OS (n=167)		RFS (n=167)	
	C-index (95% CI)	*p value*	C-index (95% CI)	*p value*
**TCF7**	0.701 (0.654-0.748)		0.654 (0.610-0.698)	
**Grade**	0.594 (0.539-0.649)		0.564 (0.511-0.617)	
**Grade+TCF7**	0.746 (0.690-0.802)	<0.001	0.686 (0.631-0.740)	<0.001
**TNM**	0.732 (0.685-0.779)		0.690 (0.646-0.734)	
**TNM+TCF7**	0.795 (0.752-0.838)	<0.001	0.740 (0.695-0.785)	<0.001
**TNM+Grade**	0.765 (0.715-0.815)		0.728 (0.678-0.777)	
**Nomogram**	0.820 (0.778-0.862)	<0.001	0.758 (0.711-0.805)	0.004

OS, overall survival; RFS, recurrence-free survival; C-index, concordance index; CI, confidence interval.

A higher c-index indicates better discrimination.

### Prognostic Nomogram of OSCC and Its Calibration Curve

**Figure 3:**
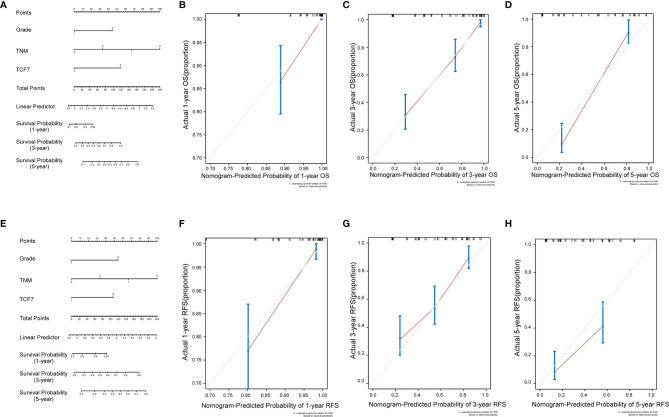
Nomograms and calibration curves of OS versus RFS. Three independent predictors, including TCF7^+^ T cells, TNM stage, and histological grouping, were incorporated into the nomogram for OS **(A)** and RFS **(E)**. Calibration curves were established for predicting OS at 1 year **(B)**, 3 years **(C)** and 5 years **(D)** for OSCC patients and RFS at 1 year **(F)**, 3 years **(G)** and 5 years **(H)** for OSCC patients. OS, overall survival; RFS, recurrence-free survival.

We incorporated the TNM stage, histological grade, and TCF7^+^ T cell percentage status into the Cox risk regression model to construct two nomograms to predict OS and RFS in OSCC patients at 1, 3, and 5 years after surgery (**Figures 3A, E**). The C-index displayed good predictive accuracy for both the OS and RFS nomograms (OS: C-index=0.728, 95% CI=0.678 to 0.777); C-index=0.758, 95% CI=0.711 to 0.858) (**Table 4**). We also constructed calibration curves for predicting OS and RFS at 1, 3 and 5 years by internal resampling 1000 times (**Figures 3B**–**D**, **F**–**H**), and the calibration curves showed good consistency in predicting and the actual event occurrence of patients.

### TCF7^+^ T Cell Expression Improves the Clinical Utility of Existing Models

**Figure 4:**
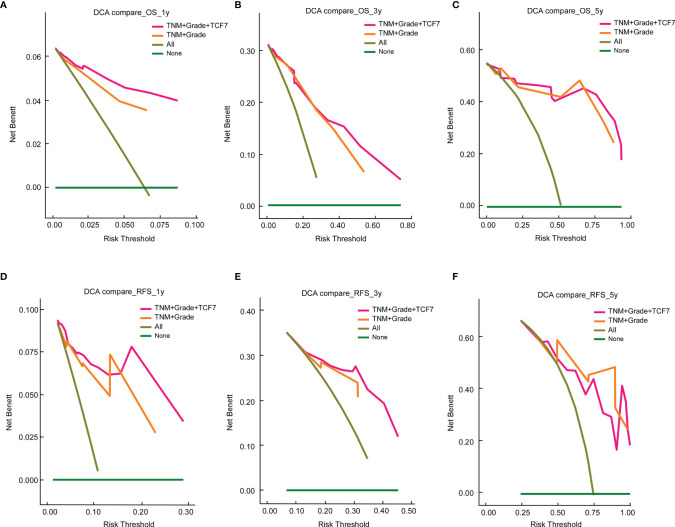
Decision curve analysis comparison of the nomogram and “TNM+Grade” models at 1 year **(A)**, 3 years **(B)**, and 5 years **(C)** for OS and 1 year **(D)**, 3 years **(E)**, and 5 years **(F)** for RFS. DCA, decision curve analysis. OS, overall survival; RFS, recurrence-free survival.

We further compared the clinical utility of models (“TNM+Grade+TCF7” vs. “TNM+Grade”) using decision curves within 1, 3 and 5 years (**Figure 4**). First, for both the “TNM+Grade+TCF7” and “TNM+Grade” prediction models, the DCA showed that the net benefit range was greater than that of the prediction model with all variables included (“All”). Second, the area under the decision curve of “TNM+Grade+TCF7” was larger than that of “TNM+Grade” at 1, 3, and 5 years for OS (**Figures 4A**–**C**) and RFS (**Figures 4D, E**) but smaller than the area under the decision curve at year 5 (**Figure 4F**). This finding also indicated that adding the TCF7^+^ T cell expression level improved the clinical utility of the existing model, suggesting that TCF7^+^ T cells warrant further research.

## Discussion

OSCC is a prevalent tumor of the head and neck cancer (HNSCC) type, and immunotherapy characterized by immune checkpoint inhibitors (ICIs) has emerged as therapy for OSCC in recent years. Because of the high tumor heterogeneity of OSCC, ICIs such as nivolumab are only beneficial for a small number of patients; indeed, ICIs generally have low response rates, with short remission durations in HNSCC (14). Hence, identifying new markers to improve existing treatment strategies is urgent.

T cell factor 1 (TCF1), encoded by TCF7, is a transcription factor of the classical Wnt/β-catenin signaling pathway (15, 16) that maintains the stem cell-like state of T cells (17) and their lymphatic recirculation and self-renewal potential (18). Kurtulus et al. (19) stated that an effective antitumor response requires TCF7/TCF1 expression; in the absence of TCF7 expression, memory precursor cells show little resistance. Plesca et al. (20) showed that an elevated frequency of TCF7^+^ T cells in melanoma infiltrates is associated with beneficial clinical outcomes in patients treated with anti-PD-1 therapy.

In the present study, we found that high TCF7 expression was associated with a better prognosis in OSCC. Additionally, high TCF7 expression was an independent predictor for both OS and RFS. We also found that TCF7^+^ T cell expression levels were strong predictors of TNM stage III+IV and a histological grade of poor differentiation. Concerning current models such as the TNM model, adding pathological grading and TCF7^+^ T cell expression levels improved the predictive power. We also generated two nomograms with a good C-index to predict OS and RFS in OSCC patients. Finally, comparison of the range of net benefits by DCA suggests that the nomogram has good clinical utility.

One of the novelties of our study is that the TCGA data were used for prediction and screening to identify target immune subgroups. We first investigated the effect of CD3 and TCF7 on the prognosis of OSCC patients starting at the transcriptional level, and four groups of patients were obtained based on high and low expression of CD3 and TCF7. Although no significant difference was found in OS among the four groups, we detected a trend that the magnitude of the probability of survival was DH group > CD3^hi^ group, TCF7^hi^ group > DL group. Therefore, we excluded the CD3^hi^ and TCF7^hi^ groups, divided the remaining 129 patients into DH and DL groups, and then performed survival analysis. We found that OSCC patients in the DH group had a better prognosis than OSCC patients in the DL group after excluding the effect of the background level (not a single CD3^hi^ expression or a single TCF7^hi^ expression effect). Thus, TCF7^+^ T cells with high expression of both CD3 and TCF7 may affect the prognosis of OSCC patients.

Additionally, the algorithm of clinical information to obtain cutoff values was applied, making the statistical results closer to clinical reality. For more convenient statistics, we converted the proportion of TCF7^+^ T cells (continuous variable) to the TCF7^+^ T cell expression level (dichotomous variable) to classify all patients into two groups. We used X-tile to calculate the optimal cutoff value of TCF7^+^ T cell expression based on survival data, and this method is convincing for use in various diseases (21–23).

To avoid the effect of X-tile grouping, we performed univariate analysis using tumor-infiltrating CD3^+^ T lymphocyte (TIL) expression and TCF7^+^ T cell expression as continuous variables. We determined no significant difference in the prognosis based on tumor-infiltrating CD3^+^ T lymphocyte (TIL) expression, which excluded the effect of the entire tumor-infiltrating CD3^+^ T lymphocyte (TIL) background, and then investigated a subpopulation of TCF7^+^ T cells. We determined that the difference in TCF7^+^ T cell expression (continuous variable) was strongly significant for prognosis in univariate analysis and then transformed TCF7^+^ T cell expression into TCF7^+^ T cell expression (dichotomous variable) for multivariate analysis and nomogram production.

Survival analysis further suggested that at two different clinicopathological risk levels,the TCF7^hi^ group at high clinical and pathological risk levels predicted a good prognosis for OSCC patients, suggesting that immune infiltration of TCF7^+^ T cells in advanced OSCC or poorly differentiated OSCC may improve patient survival. This finding may be related to precursor exhausted T cells. Exhausted T cells can be classified into precursor exhausted T cells and terminally differentiated exhausted T cells according to their phenotype (24). Precursor exhausted T cells have low expression of exhaust surface markers (25–27), and TCF1/TCF7 is a key regulator of precursor exhausted T cells (25). Compared with terminally differentiated exhausted T cells, precursor exhausted T cells have stronger proliferative capacity (17) and differentiation potential (28) and longer longevity (29). Precursor exhausted T cells also exhibit antitumor effects in patients with melanoma (10), pancreatic cancer (30) and colorectal cancer (31).

To better evaluate the prediction accuracy of the model, we used the index of concordance (C-index). In the multivariate Cox risk regression model, we clarified that the inclusion of the TCF7^+^ T cell percentage improved the prediction accuracy of existing accepted models by comparing the difference in the C-index. To improve the clinical usefulness of the prediction model, we transformed the modified model into a nomogram. The nomograms were resampled 1000 times for internal validation, yielding calibration curves with good agreement. To assess clinical utility, we used DCA to compare the area under the curve (net benefit range), and the inclusion of the TCF7^+^ T cell percentage improved the clinical utility of the existing model.

However, this study has limitations. The specific mechanism by which TCF7^+^ T cells affect tumor prognosis is currently unclear, warranting further investigation in combination with single-cell sequencing and other technologies to explore the specific mechanism of TCF7. Additionally, support is needed from external validation sets in the risk Cox regression model. Therefore, further studies are needed to investigate the role of TCF7^+^ T cells in OSCC.

## Conclusion

The current study showed that the TCF7^+^ T cell expression level can be used as an independent predictor for OSCC patients and that the nomogram incorporating the TCF7^+^ T cell expression level has good discrimination, calibration and clinical utility; however, it should be externally validated. TCF7^+^ T cells are expected to serve as new immunotherapeutic markers and should be further validated by basic experiments.

## Data Availability Statement

The original contributions presented in the study are included in the article/**Supplementary Material**, further inquiries can be directed to the corresponding authors.

## Ethics Statement

The studies involving human participants were reviewed and approved by Institutional Review Board of the Sun Yat-Sen Memorial Hospital, Sun Yat-sen University. The patients/participants provided their written informed consent to participate in this study.

## Author Contributions

HR: Data curation; Formal analysis; Methodology; Writing original draft; Writing-review & editing. TC and XW: Data curation; Formal analysis; Methodology, Writing-original draft. YP: Data curation; Formal analysis; Project administration. TL: Resources; Validation. ZO: Supervision, Resources. LQ: Methodology, Formal analysis. QL and LZ: Supervision. HL: Resources. FW: Project administration. SR and ZL: Methodology. SF: Conceptualization; Supervision; Writing-original draft; Writing-review & editing. JL: Conceptualization; Funding acquisition; Supervision; Writing original draft; Writing-review & editing. All authors contributed to the article and approved the submitted version.

## Funding

This study was supported by the Natural Science Foundation of China (Grant Nos. 82072990, 81872194 and 81772890), grants from Guangzhou Science and Technology Bureau (2021A1515012355), Science and Technology Program of Guangzhou Grant numbers: 2021020104207, and Open Project Contract (2020B1212060018OF003) from Guangdong Provincial Key Laboratory of Malignant Tumor Epigenetics and Gene Regulation. The funders had no role in the study design, data collection and analysis, decision to publish, or manuscript preparation.

## Conflict of Interest

The authors declare that the research was conducted in the absence of any commercial or financial relationships that could be construed as a potential conflict of interest.

## Publisher’s Note

All claims expressed in this article are solely those of the authors and do not necessarily represent those of their affiliated organizations, or those of the publisher, the editors and the reviewers. Any product that may be evaluated in this article, or claim that may be made by its manufacturer, is not guaranteed or endorsed by the publisher.

## Supplementary Material

Supplementary Figure 1
**(A)** A total of 212 patients were divided into four groups for Kaplan–Meier analysis. **(B)** A total of 129 patients were divided into two groups for Kaplan–Meier analysis. Green=CD3^hi^ TCF7^hi^ (DH). Red=CD3^low^ TCF7^low^ (DL). Blue=CD3^hi^ TCF7^low^. Purple=CD3^low^ TCF7^hi^. Survival curves were compared by the log-rank test. *p*<0.05 was considered to indicate significance.
