# Supplementary figures and images for: Correlation Between TCF7+ T Cells and Prognosis of Patients With Oral Squamous Cell Carcinoma
Source: Front Oncol. 2022 Mar 8;12:782058. doi: 10.3389/fonc.2022.782058 (PMC8957207; doi:10.3389/fonc.2022.782058)

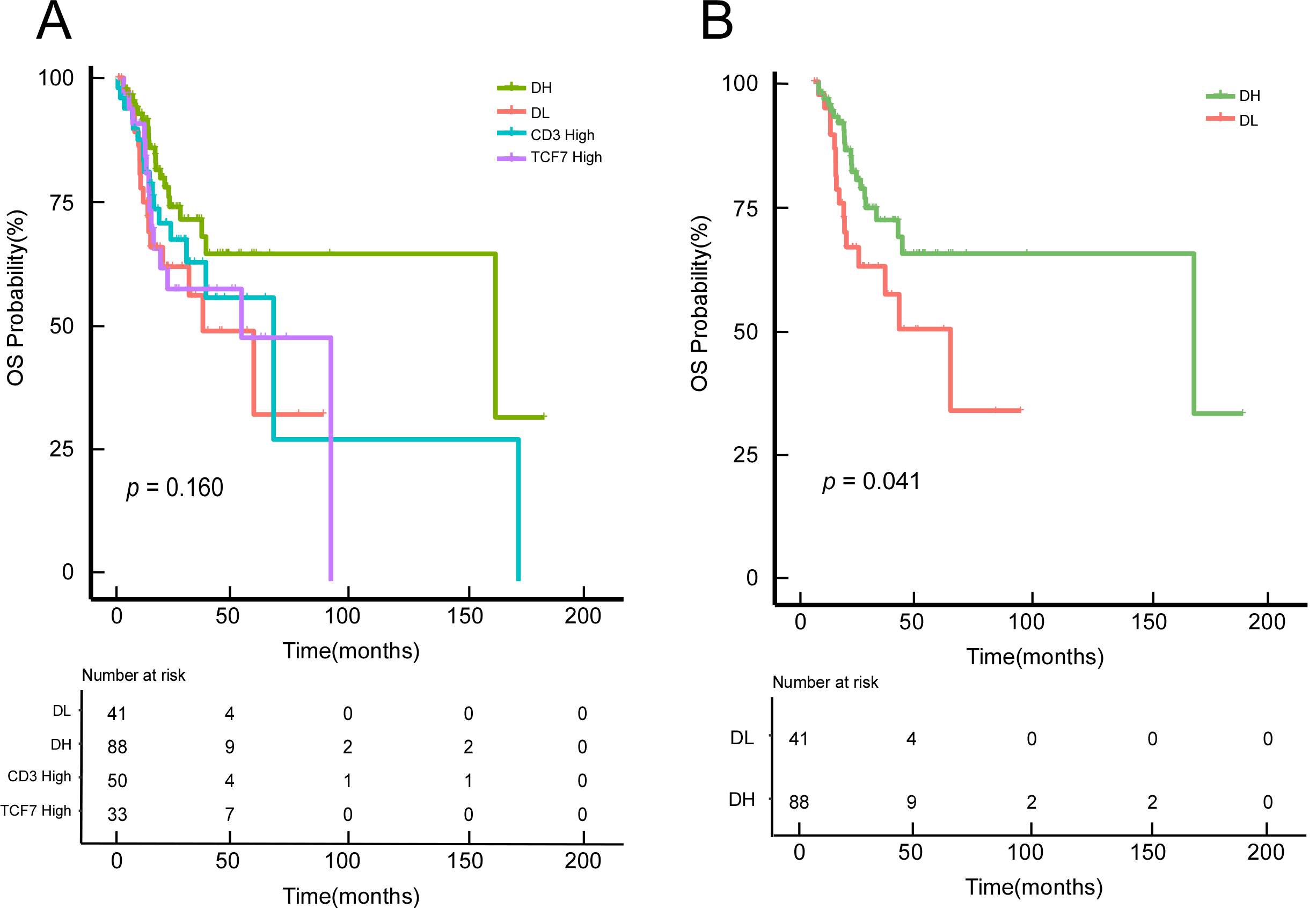

Supplement: Supplementary Figure 1 — (A) A total of 212 patients were divided into four groups for Kaplan–Meier analysis. (B) A total of 129 patients were divided into two groups for Kaplan–Meier analysis. Green=CD3hi TCF7hi (DH). Red=CD3low TCF7low (DL). Blue=CD3hi TCF7low. Purple=CD3low TCF7hi. Survival curves were compared by the log-rank test. p<0.05 was considered to indicate significance. [file Image_1.tif]
